# Decoding the Molecular Mechanisms of Menthol Isomer Perception Based on Computational Simulations

**DOI:** 10.3390/foods14142494

**Published:** 2025-07-16

**Authors:** Mengxue Wang, Fengge Wen, Lili Zhang, Baoguo Sun, Jianping Xie, Shihao Sun, Yuyu Zhang

**Affiliations:** 1 Beijing Life Science Academy, Beijing 102209, China; sherry_1182@163.com (M.W.); 13949989546@139.com (F.W.); xiejp@blsa.com.cn (J.X.); 2 College of Food Science and Engineering, Tianjin University of Science and Technology, Tianjin 300457, China; 3 Key Laboratory of Geriatric Nutrition and Health, Beijing Technology and Business University, Ministry of Education, Beijing 100048, China; zll@btbu.edu.cn (L.Z.); sunbg@btbu.edu.cn (B.S.)

**Keywords:** menthol, isomers, sensory evaluation, detection threshold, molecular docking

## Abstract

The flavor characteristics, perception, and molecular mechanisms of eight menthol isomers were investigated by sensory analysis combined with computational simulations. The sensory analysis results show significant differences in the odor profiles of the different menthol isomers. Among them, L-menthol shows a pleasant, sweet, and mint-like odor with a distinct freshness and no off-flavors, whereas the remaining seven isomers were interspersed with negative odors (musty, herbal, or earthy aromas). L-menthol and D-menthol had the lowest detection thresholds of 5.166 and 4.734 mg/L, respectively. The molecular docking results of the menthol isomers with olfactory receptors (Olfr874, OR8B8, and OR8B12) indicate that hydrogen bonding and hydrophobic interactions were the key binding forces. The binding energy ranged from −7.3 to −5.1 kcal/mol. Residues His-55 (Olfr874), Thr-56 (Olfr874), Leu-55 (OR8B8), Tyr-94 (OR8B8), Thr-57 (OR8B8), Phe-199 (OR8B12), and Ser-248 (OR8B12) with high frequencies particularly contributed to the recognition of menthol isomers. These findings contribute to a deeper understanding of the olfactory perception mechanism of menthol and provide data support for the development and precise application of minty odorants.

## 1. Introduction

Menthol is a naturally occurring volatile cyclic terpene alcohol with pharmacological effects, such as anti-inflammatory, antimicrobial, and antiviral properties, and it has been widely used in the production of cosmetics, pharmaceuticals, and food flavors [1,2]. It is characterized by a minty aroma and freshness, and is effective as an immunomodulator against cognitive decline in Alzheimer’s disease mice [3]. Menthol is known to be a TRPM8 agonist, and its mechanism of activating the TRPM8 channel at the atomic level was clarified by Xu et al. [4]. In 2015, the olfactory receptor MOR161-2 (also known as Olfr874) in *Mus musculus* was identified to be responsive to L-menthol, and the specificity of MOR161-2 for the enantiomeric recognition of menthol was demonstrated at the cellular level [5]. However, the unknown structure and function of the olfactory receptor have resulted in a lack of understanding of the molecular mechanism involved in menthol recognition by the olfactory receptor.

The structure of odorants is closely related to their olfactory properties, and stereoisomers tend to differ in their odor characteristics and detection thresholds. Research shows that an orchid-like fragrance is a characteristic of high-quality tea, and its formation is directly related to the molecular structure of methyl jasmonate. *cis*-Methyl jasmonate is considered the main contributor to the “orchid-like fragrance” of tea, while other isomers of this compound do not play a significant role in this characteristic [6]. Linalool in Meitan Cuiya tea has a floral and lavender aroma, while its enantiomers exhibit distinct aroma profiles: (*S*)-linalool exhibits a sweet, floral aroma, while (*R*)-linalool displays woody notes [7]. Similarly, (*R*)-limonene from tea has an orange-like aroma, while (*S*)-limonene presents a pungent turpentine odor. In addition, (*R*)-*α*-terpineol has a strong lilac aroma, while its enantiomer (*S*)-*α*-terpineol exhibits a coniferous odor [8]. Menthol, as a compound with three chiral carbon centers, has eight optical isomers (L/D–menthol; L/D–neomenthol; L/D–isomenthol; L/D–neoisomenthol), among which L-menthol has a distinctive mint flavor, while the other isomers have distinctive musty or other undesirable odors [9,10,11]. Therefore, elucidating the structure–activity relationships of menthol isomers is important for the design and development of novel mint aroma compounds.

The diversity and complexity of olfactory receptor gene expression are closely related to the ecological needs of species in their living environment. The stability of conserved olfactory gene expression ensures the maintenance and continuation of olfactory functions among species, whereas non-conserved olfactory genes confer unique chemical perception to each species [12,13]. Thus, the coexistence of conserved and non-conserved genes directly influences the species’ perceptual experience of a compound’s aroma. According to the National Center for Biotechnology Information database https://www.ncbi.nlm.nih.gov/IEB/Research/Acembly/av.cgi?db=mouse&c=gene&a=fiche&l=Olfr874 (10 November 2024), OR8B8 and OR8B12 are the homologous receptors of Olfr874 in humans. Currently, existing research on OR8B8 mainly focuses on its expression positions, with its ligands receiving limited attention. For example, in addition to being expressed in the tongue [14], OR8B8 expression has been detected in many types of tumors, such as breast cancer [15,16,17]. Similarly, there are few reports on OR8B12. Currently, it is known that OR8B12 can be activated by indole (100 μM) [18]. Additionally, OR8B12 is also considered a specific protein associated with Alzheimer’s disease [19]. However, it is not clear whether there are differences in the aroma recognition of menthol across species. Recently, advances in computer simulation techniques have provided technical support for resolving interactions between odorants and olfactory receptors in the absence of specific structural and functional data, helping to reveal the olfactory perception mechanisms of various aroma compounds [20]. Molecular docking simulations and physical modeling revealed that the maximal olfactory response activity of cow btOR9Q2 to 4-methylphenol is 10 times higher than that of human hOR9Q2 [21]. Zhu et al. elucidated the interactions of key lactone chiral compounds (γ-octalactone and γ-undecalactone) with olfactory receptors (OR1A1 as an example) in Longjing tea using molecular docking and molecular dynamics [22]. Methods to simulate cinnamon aroma have also been developed using molecular docking screening and odor activity value (OAV) analysis [23]. Therefore, employing molecular docking techniques to study the molecular interaction mechanisms between menthol and species-specific olfactory receptors, while clarifying the contributions of conserved and non-conserved residues to menthol binding, is crucial for revealing interspecies differences in menthol perception from the perspective of diversity in homologous gene expression.

In this study, the olfactory stimulation characteristics of menthol isomers were investigated through sensory analysis, and the dose–effect relationships between the concentration and its aroma perception were clarified. The action modes between menthol isomers and olfactory receptors were analyzed using the molecular docking technique. The diversity of menthol-specific olfactory receptor genes in different species was clarified based on sequence alignment, and the molecular mechanism of menthol recognition by olfactory receptors in different species was further investigated. This study aims to explore the olfactory perception mechanism of menthol at the molecular level, and it provides new ideas for the development of novel mint aroma compounds.

## 2. Materials and Methods

### 2.1. Materials and Chemicals

L-menthol (99.0%; CAS No. 2216-51-5) and D-menthol (99.0%; CAS No. 15356-60-2) were purchased from Macklin (Shanghai, China). D-isomenthol (99.5%; CAS No. 23283-97-8) was purchased from Anpel (Shanghai, China). L-neomenthol (95.5%; CAS No. 2216-52-6), L-neoisomenthol (99.8%; CAS No. 20752-34-5), and D-neoisomenthol (99.4%; CAS No. 64282-88-8) were purchased from CATO (Beijing, China). D-neomenthol (99.8%; CAS No. 20747-49-3) and L-isomenthol (99.7%; CAS No. 20752-33-4) were purchased from TLC (Beijing, China). Liquid paraffin (CAS No. 8042-47-5) was purchased from Macklin (Shanghai, China).

### 2.2. Sensory Analysis

#### 2.2.1. Recruitment and Training of the Panelists

Sensory experiments were conducted using the methods of Xiao and Jiang et al., with minor modifications [24,25,26]. An application detailing the feasibility and controllability of the experiment was submitted to and approved by the Ethics Committee of Beijing Technology and Business University (BTBU202333, Beijing, China, 3 2023–3 2026) prior to the start of the experiment. Ten experienced sensory panelists (five males and five females, aged 20–30 years) were recruited from the Key Laboratory of Flavor Science of China General Chamber of Commerce (Beijing, China). All panelists, who were in good health and free of sensory defects, were provided with a thorough explanation of the requirements and the potential risks of the study, and they participated voluntarily and reserved the right to withdraw from the study at any time. In order to ensure the reliability of the sensory assessment, all panelists received three 30 min pre-test training sessions to familiarize themselves with the use of the scale. After the training sessions, the panelists were tested to identify and rank the intensity of seven odor profiles (sweetness, mint flavorings, freshness, earthy aromas, herbal, woody, and musty) in a sensory chamber at approximately 20 °C, and all passed the assessment.

#### 2.2.2. Sensory Quantitative Descriptive Analysis

Menthol isomer solutions were prepared by dissolving the standards separately in colorless and odorless liquid paraffin. Subsequently, serial dilutions of menthol isomer solutions were carried out using a dilution factor of 2. The concentrations of the solutions used for sensory evaluation were set at 3.125 ×10^−4^~5.000 × 10^−3^ mg/L. Dilutions with different concentrations were transferred to brown sample bottles and vortexed for sensory evaluation. The panelists were instructed to avoid spicy foods or the use of strong-smelling personal care products on the day of testing. During the sensory evaluation, the panelists were asked to describe the aroma attributes of the provided samples and to score them according to odor intensity. A 10-point score was used, with a score of 1 indicating weak odor and a score of 10 indicating very strong odor. The odor intensity showed an increasing trend from 1 to 10. The test was repeated three times for all samples, and the sensory scores were retained to one decimal place. To prevent interference, panelists were allowed a 5 min break between different samples. During the experiment, the samples were provided to panelists in a randomized order to ensure evaluation anonymity.

#### 2.2.3. Determination of Odor Detection Thresholds

The detection thresholds of menthol isomers were determined using the three-alternative forced-choice (3-AFC) procedure, according to GB/T 22366-2022 [27]. Each of the eight menthol isomers was dissolved in colorless, odorless liquid paraffin and diluted in a gradient with a dilution factor of 10 using 5.000 × 10^−3^ mg/L as the highest concentration solution. During the sensory evaluation, three samples were simultaneously provided to the panelist, one of which was a test sample containing a menthol stimulus, and the other two were reference samples (liquid paraffin). The prepared samples are provided to the team members in the inspection area, who evaluate the samples according to the sensory analysis method outlined in GB/T 22366-2022 [27]. The samples were randomly coded with three digits and arranged such that the first sample was placed at the front left of the panelist, with the remaining samples arranged sequentially from front to back. The arrangement was recorded on the answer sheet as well. An arrangement of BAA, ABA, and AAB was adopted to balance the order of the three samples provided in front of the panelist in order to avoid positional deviation. A 5 min break was allowed between samples to avoid olfactory fatigue. Panelists were asked to sniff three samples and identify the one that differed. If a solution containing the menthol isomer was identified, the same test was repeated with the next set of samples at lower concentrations. The results of the panelists’ judgment were recorded, and the probability of detection was calculated. Using the correction formula, the corrected probability of detection was calculated, and the correction formula was P = (3p − 1)/2, where p is the correct response rate at each concentration, and P is the probability corrected for chance effects [28]. The detection threshold can be defined as the concentration value corresponding to a 50% probability of correct detection. Origin 2021 software (OriginLab, Northampton, MA, USA) was used to plot the data using the S-curve method, and at ordinate P = 0.5, the corresponding abscissa represents the threshold.

### 2.3. Molecular Docking

#### 2.3.1. Preparatory Work

Receptor protein PDB files (OR8B8: Q15620, OR8B12: Q8NGG6, Olfr874: Q7TRE6) were obtained from the UniProt https://www.uniprot.org/ (15 December 2024) database. The SDF files for the eight menthol isomers were obtained from the PubChem database and converted to PDB format using the Open Babel GUI 2.3 software https://openbabel.org/docs/index.html (15 December 2024)

#### 2.3.2. Model Construction and Optimization

Redundant water molecules and small-molecule ligands were removed from the receptor protein using PyMOL (DeLano Scientific LLC, South San Francisco, CA, USA), and the receptor protein was imported into the AutoDock Vina 1.2.0 program (Scripps Research, La Jolla, CA, USA) for hydrogenation and charge calculation and saved in “pdbqt” format.

#### 2.3.3. Molecular Docking

The interactions between the receptor and menthol isomers were analyzed using the AutoDock Vina 1.2.0 program (Scripps Research, La Jolla, CA, USA), and the optimal conformation was generated in the predicted docking pocket. After docking, the best conformational pair was selected based on the docking score. Discovery Studio 2019 Client software was utilized to generate two-dimensional (2D) maps of receptor–ligand interactions, while the optimal docking files were imported into PyMOL software (DeLano Scientific LLC, South San Francisco, CA, USA) to obtain three-dimensional (3D) maps of receptor–ligand interactions. Finally, the molecular docking results were organized and analyzed for the minimum binding energy, key binding pockets and sites, main forces, etc.

## 3. Results and Discussion

### 3.1. Aroma Profiles of Eight Menthol Isomers

**Figure 1:**
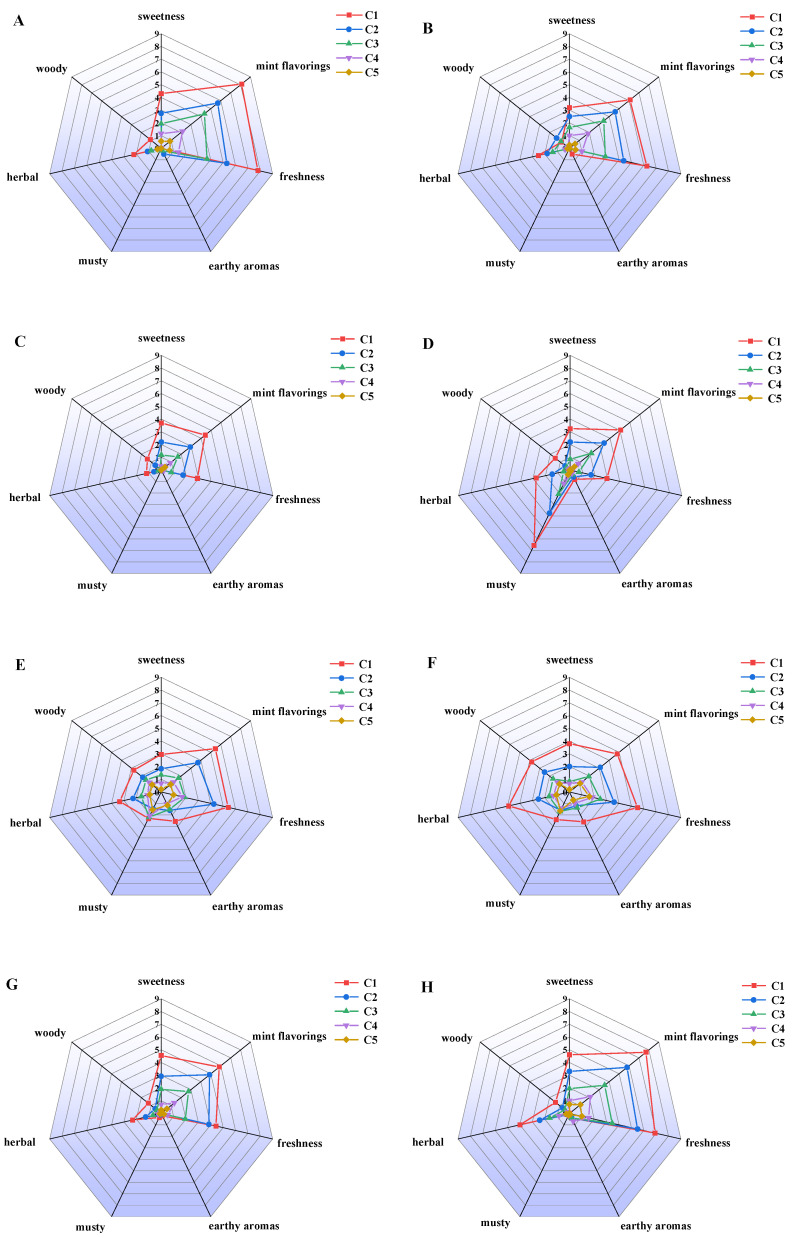
The radar plots of the sensory evaluation of eight menthol isomers. (**A**): L-menthol; (**B**): D-menthol; (**C**): L-neomenthol; (**D**): D-neomenthol; (**E**): L-isomenthol; (**F**): D-isomenthol; (**G**): L-neoisomenthol; (**H**): D-neoisomenthol. C1–C5: The five concentrations of the sample (from high to low).

The aroma characteristics of the eight menthol isomers are shown in Figure 1. L-menthol is known to be a colorless and transparent needle-like crystal with a cool mint flavor. In the present study, L-menthol (5.000 × 10^−3^ mg/L, C1) exhibited the most prominent mint flavor and freshness, with a hint of sweetness and herbal character (Figure 1A), giving an overall impression of a penetrating but not long-lasting aroma. D-menthol, as an enantiomer of L-menthol, had a similar aroma profile to L-menthol at the concentrations tested, but the intensity of its predominantly mint flavor and its freshness were weaker than those of L-menthol (e.g., C1 5.000 × 10^−3^ mg/L, C2 2.500 × 10^−3^ mg/L) (Figure 1B). In addition, in the sensory evaluation of the two isomers, from the results of the sensory evaluation, it was evident that the two isomers possessed a slight sweetness and herbal odor (Figure 1A,B). The flavor profile of a compound is closely related to the spatial consistency of its stereoisomers [29,30]. Differences in the specific binding of the odorant to olfactory receptors resulting from minor structural differences could induce significant changes in aroma [31,32]. Compared to L-menthol, L-neomenthol showed a marked change in its aroma profile, being much weaker in terms of its minty notes and freshness (Figure 1C). Notably, D-neomenthol differed from its enantiomer L-neomenthol in its flavor profile, having a prominent herbal and musty odor in addition to a similar sweetness, minty notes, and freshness as L-neomenthol, and the intensity of its musty odor was the highest among the eight stereoisomers at the same concentrations (Figure 1D). L-isomenthol and D-isomenthol had lower intensities with respect to their mint character and freshness compared to L-menthol, and both compounds exhibited a combination of sweet, woody, herbal, musty, and earthy aroma profiles (Figure 1E,F). In the food industry, racemic mixtures of chiral compounds, such as menthol, are frequently added to plant extracts to enhance the natural flavor of some foodstuffs [33]. Menthol isomers and their derivatives are widely used in organic synthesis as starting materials and as chiral auxiliaries. They have also been frequently employed as flavorings and drugs in medicinal preparations [34]. D-isomenthol has a similar aroma characteristic to L-isomenthol. It is worth mentioning that in addition to their favorable aroma profile, other unfavorable odors, such as woody, earthy, and musty odors, were highlighted in this pair of enantiomers but not in the other three pairs of enantiomers (Figure 1F). The aroma profile of L-neoisomenthol was similar to that of L-neomenthol, but its mint and herbal character and freshness were slightly stronger than those of L-neomenthol at the same concentrations (Figure 1G). For D-neoisomenthol, the aroma profile was most similar to that of L-menthol, and the intensity of the minty freshness was comparable at the same concentrations; however, the intensity of the herbal aroma in this compound was substantially higher than that in L-menthol at the same concentration (Figure 1H).

Overall, the eight menthol isomers presented different odor characteristics (Figure 1). L-menthol had a pronounced minty aroma and freshness, characterized by pleasant sweetness and a lower intensity of negative odors, whereas the remaining seven isomers present negative odors, such as musty, herbal, or earthy aromas, to varying degrees, which is consistent with the existing literature [35,36]. For the other seven isomers, the mint flavoring of D-neoisomenthol was the most pronounced and most similar to that of L-menthol, followed by D-menthol and L-neoisomenthol, while the intensity of the minty aroma of D-neomenthol and L-isomenthol was reduced under the same concentration conditions. The minty aroma of D-isomenthol and L-neomenthol was the least pronounced. The freshness of D-neoisomenthol was the most pronounced among the seven isomers at the same concentration, except for L-menthol, followed by D-menthol, L-isomenthol, D-isomenthol, and L-neoisomenthol, while L-neomenthol and D-neomenthol showed significantly lower cooling intensities. The distinct compositions and ratios of menthol isomers in different species of mint plants, combined with the diverse aroma characteristics of menthol isomers, contribute to the unique flavor of each type of mint [33]. In addition, the aroma intensity of menthol isomers was observed to diminish as the concentration of the solution decreased. However, as the concentration was gradually reduced, both the pleasant minty aroma and coolness and the undesirable woody and musty odors were gradually weakened until they were almost undetectable. This reduction affects the aromatic intensity of the menthol isomers and alters the complexity and layering of their aroma, causing the intense aroma to become progressively more homogeneous and fainter.

### 3.2. Threshold Analysis of Eight Menthol Isomers

**Figure 2:**
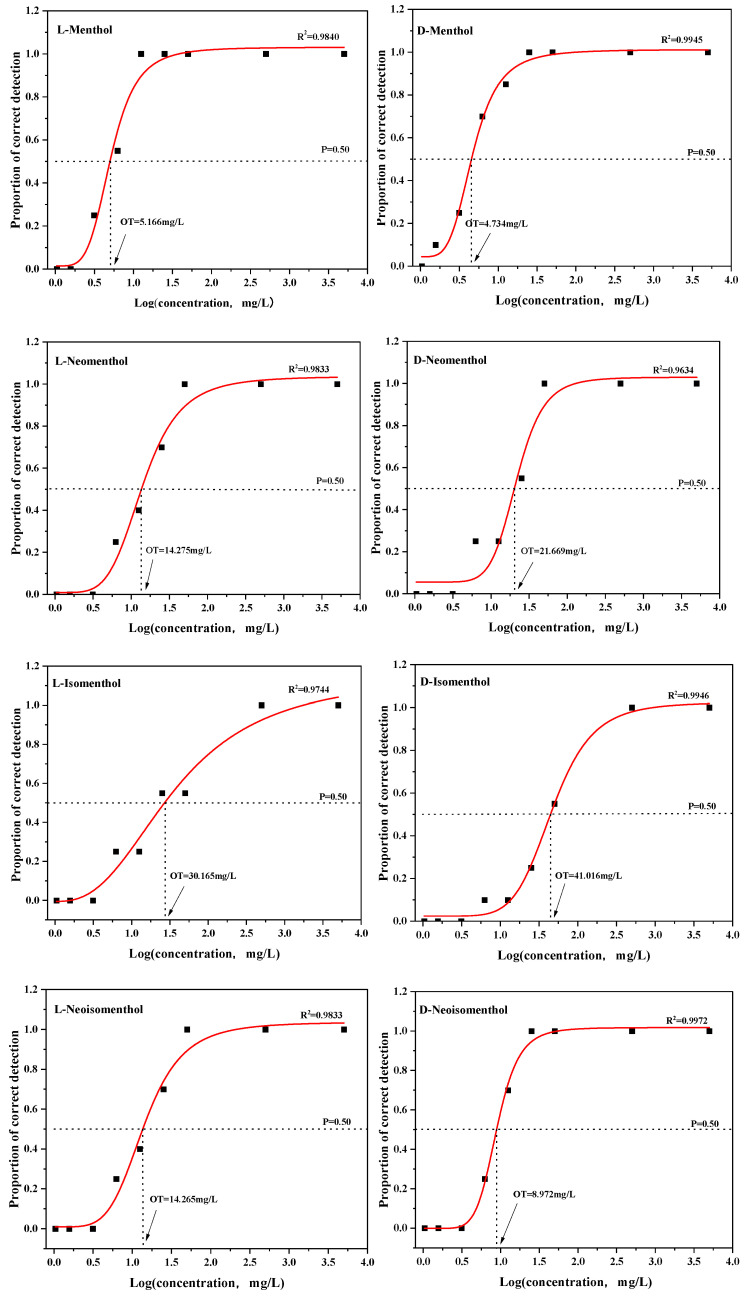
Detection thresholds of eight menthol isomers. The vertical axis represents the probability of correct detection of odors by test subjects, and the horizontal axis represents the logarithm of odor concentration.

The olfactory threshold is an important indicator of a compound’s contribution to flavor [37]. Existing data on the detection threshold of L-menthol were mainly obtained by analyzing tobacco matrices (315 ppm) [38,39]. In addition, 1.8 mg of menthol in 1 g of flavored tobacco was detectable during sensory evaluation [40], and the threshold of perception for L-menthol in liquid paraffin (a colorless, odorless organic solvent) was 4.734 mg/L. In another study, a combination of sensory and chemical analyses determined that the sensory difference threshold of menthol odor in flavored tobacco corresponded to 43 (37–50)% menthol-flavored tobacco blends, and 1.8 (1.6–2.1) mg of menthol per gram of tobacco was detected [40]. The physical properties of the pure enantiomers of menthol and the racemic mixtures are slightly different [41]. More studies have been carried out on racemic menthol and L-menthol mixtures, while fewer studies have been carried out on the other seven isomers of menthol. In this study, the thresholds of eight menthol isomers were examined using colorless and odorless liquid paraffin as a matrix, and the results are shown in Figure 2.

Among these eight menthol isomers, the thresholds ranged from 4.734 to 41.016 mg/L, with a nearly 10-fold difference between the lowest and highest thresholds, which also illustrates that slight structural differences between isomers result in dramatic changes in sensory properties. Among the eight compounds, L-isomenthol and D-isomenthol had the highest perception thresholds of 30.165 mg/L and 41.016 mg/L, respectively, which is consistent with their weaker aroma intensity. The threshold of the detection of D-neomenthol was 21.669 mg/L. The threshold for L-neomenthol was 14.275 mg/L, while the threshold for L-neoisomenthol was strikingly similar to that of L-neomenthol at 14.265 mg/L. The threshold for D-neoisomenthol was significantly smaller than that of its enantiomers at 8.972 mg/L. The detection thresholds of L-menthol and D-menthol were 5.166 mg/L and 4.734 mg/L, respectively, which were substantially lower than those of the other menthol isomers. In addition, L-menthol and D-menthol had close detection thresholds, and their aroma profiles were similar, which might be related to the similarity of their structures.

These results may be due to differences in perception thresholds as a consequence of different matrices. One study suggests that the thresholds of volatile compounds characteristic of tomato aroma were different by as much as 40-fold (2-pentenal) between a deodorized tomato homogenate medium, an ethanol/methanol/water solution, and water [42]. Specifically, acidic and basic matrices may alter the chemical structure of menthol, whereas aqueous and oily matrices may affect the diffusion rate [43].

### 3.3. Analysis of Interactions Between ORs and Menthol Isomers by Molecular Docking

#### 3.3.1. Binding Regions Between ORs and Menthol Isomers

The interaction between odorants and olfactory receptors constitutes the initial stage of the odor detection process [44]. In humans, olfactory receptors consist of approximately 400 coding genes, constituting the largest subfamily of class A G-protein-coupled receptors (GPCRs) [45]. In the present study, the interactions of Olfr874—the specific receptor for L-menthol in *Mus musculus*—and its orthologous proteins in *Homo sapiens*, OR8B8 and OR8B12, with menthol isomers were explored. These receptors consist of 310–311 amino acids, and each transmembrane structural domain fragment consists of 17–31 amino acids.

**Figure 3:**
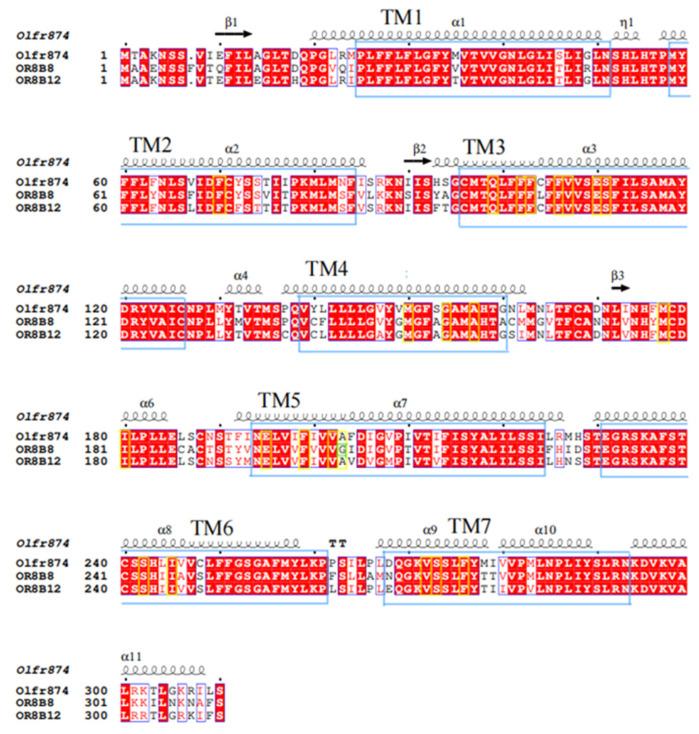
Multiple sequence comparison between mouse Olfr874 and direct human homologs OR8B8 and OR8B12. The 22 amino acid positions predicted by Man et al. [46] are marked with yellow boxes, and amino acid residues with <100% conservation are marked with green boxes. Transmembrane helices (TMHs) are indicated as large blue boxes.

In order to investigate the conserved and non-conserved nature of olfactory receptors, we performed multiple sequence comparisons of the murine-derived olfactory receptor Olfr874 and its human-derived immediate homologs, olfactory receptors OR8B8 and OR8B12, using ESPript 3.0 https://espript.ibcp.fr/ESPript/cgi-bin/ESPript.cgi (15 March 2025). Previously, Man et al. identified 22 amino acid positions that constitute the putative, broadly defined, and conserved odorant-binding pockets in the immediate homologs of the olfactory receptors [46]. Typically, functional sites with more highly conserved positions are also more conserved in their amino acid type [47]. Among these positions, we found one amino acid difference, located at Gly-204 (Figure 3).

As shown in Figure 3, OR8B8, OR8B12, and Olfr874 exhibit a certain degree of similarity in their amino acid sequences, which may reflect their shared evolutionary ancestry or similar functional requirements. However, there are also significant differences between them, which may be related to their respective specific ligand recognition capabilities. For example, OR8B12 can recognize indole [18], while Olfr874 is a specific receptor for L-menthol [5]. From a structural domain perspective, these three olfactory receptor proteins all possess the typical seven transmembrane domains characteristic of G-protein-coupled receptors. Among them, OR8B8 and OR8B12 exhibit high conservation in the transmembrane regions (especially TM1, TM3, and TM6), suggesting that they may recognize similar ligands. Among the 22 key amino acids predicted by previous studies [46], OR8B8 differs from OR8B12 and Olfr874 in one amino acid: Gly-204. This may account for their differing binding characteristics. Based on sequence alignment results, we speculate that regions with high similarity may indicate shared fundamental functions among olfactory receptors, such as the initial recognition of specific categories of odor molecules. In contrast, regions with significant differences may confer unique ligand selectivity to olfactory receptors, enabling them to perform distinct roles in olfactory perception. OR8B8 and OR8B12 belong to the same gene cluster in the human genome, while Olfr874 is their homologous gene in mice. By comparing the amino acid sequences of the three olfactory receptors, it was found that they exhibit a high degree of amino acid sequence conservation. The potential effects of the amino acid difference at site 204 (glycine vs. alanine) were analyzed. Position 204 is located at the edge of the binding pocket, and the Gly/Ala difference may regulate ligand affinity or selectivity through indirect effects (e.g., altering the orientation of adjacent key residues). Additionally, although the Gly/Ala difference at position 204 is not listed as a key site, it may subtly adjust the shape or electrostatic distribution of the binding pocket, resulting in differences in ligand binding modes or affinity. This also explains why certain isomers exhibit lower binding affinity for OR8B8. In docking analysis, residues such as His-55 (Olfr874_L-Isomenthol), Tyr-94 (OR8B8_L-Neomenthol), and Ser-248 (OR8B12_L-Isomenthol) were identified as key residues, but they exhibit differences between different species, reflecting the non-conserved nature of the sequence.

The conservation of these amino acid sites shows how homologous protein sequences differ across species. Among them, Val-108 and Val-203 can be predicted not only via sequence comparison but also via molecular docking to further verify their conservation. Gly-204, on the other hand, was predicted as a non-conserved site only via sequence comparison. These findings suggest that although certain amino acid sites have shown some non-conservation during evolution, they may still have an important impact on the function of the odorant binding pocket. By combining sequence comparison and molecular docking analyses, the role of these sites in odorant receptor function can be more fully understood.

**Figure 4:**
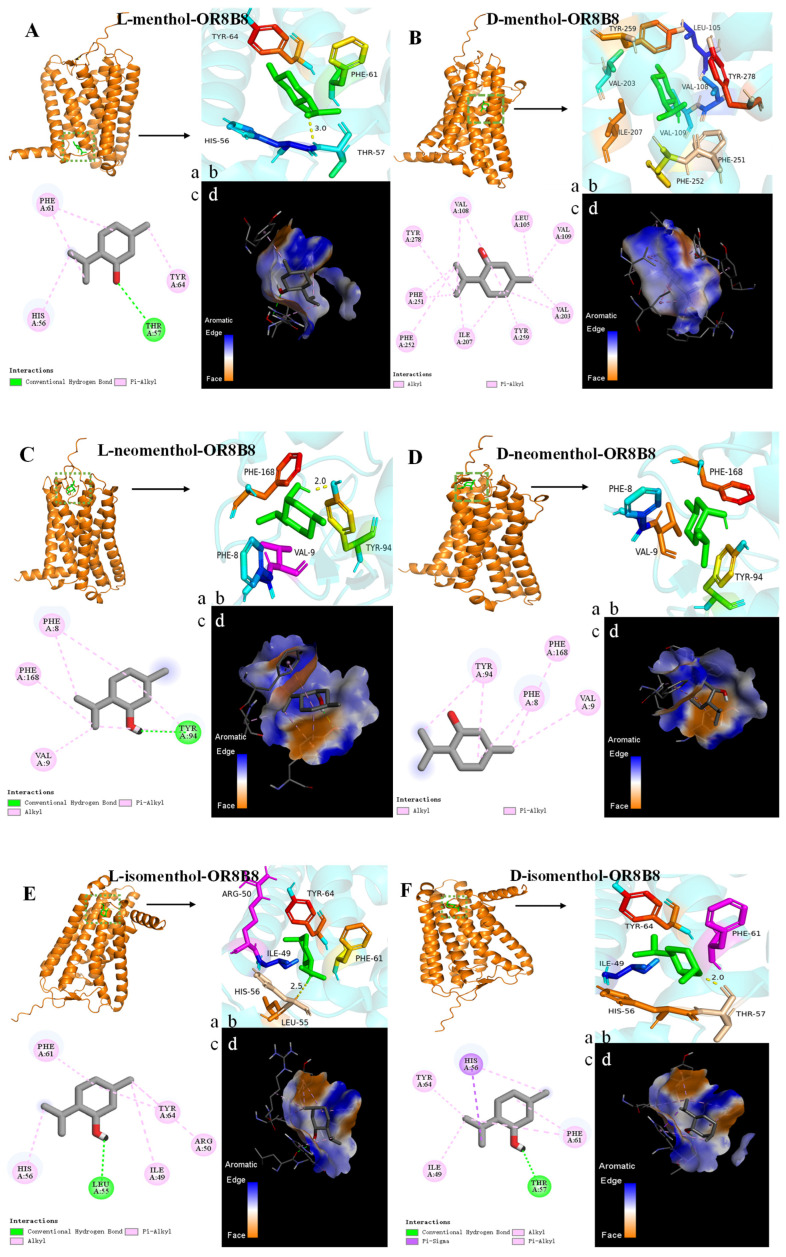

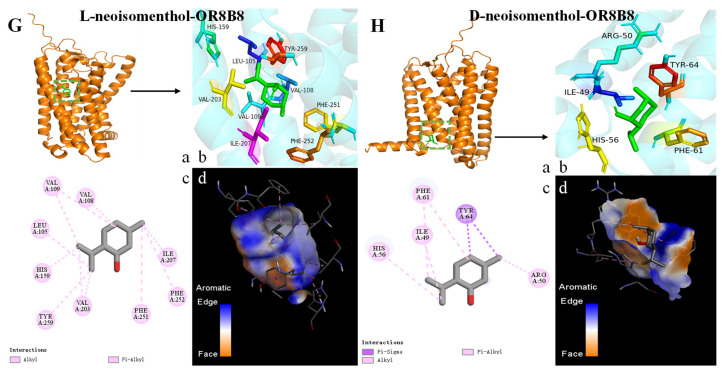
Molecular docking results of the olfactory receptor OR8B8 with eight menthol isomers. (**A**): L-menthol; (**B**): D-menthol; (**C**): L-neomenthol; (**D**): D-neomenthol; (**E**): L-isomenthol; (**F**): D-isomenthol; (**G**): L-neoisomenthol; (**H**): D-neoisomenthol. a–c: The global, local enlarged, and 2D plots of the docking. d: Binding pocket.

**Figure 5:**
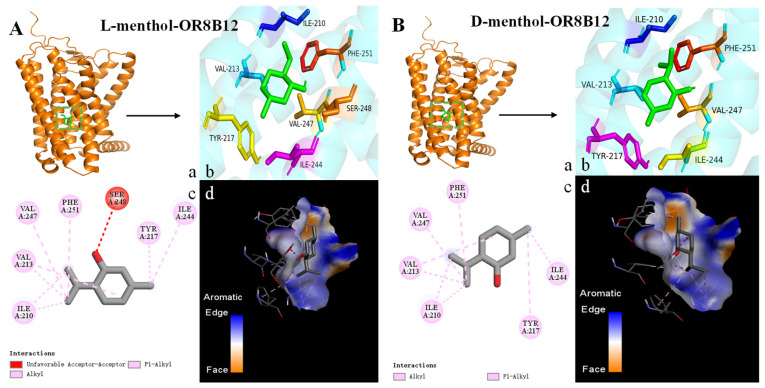

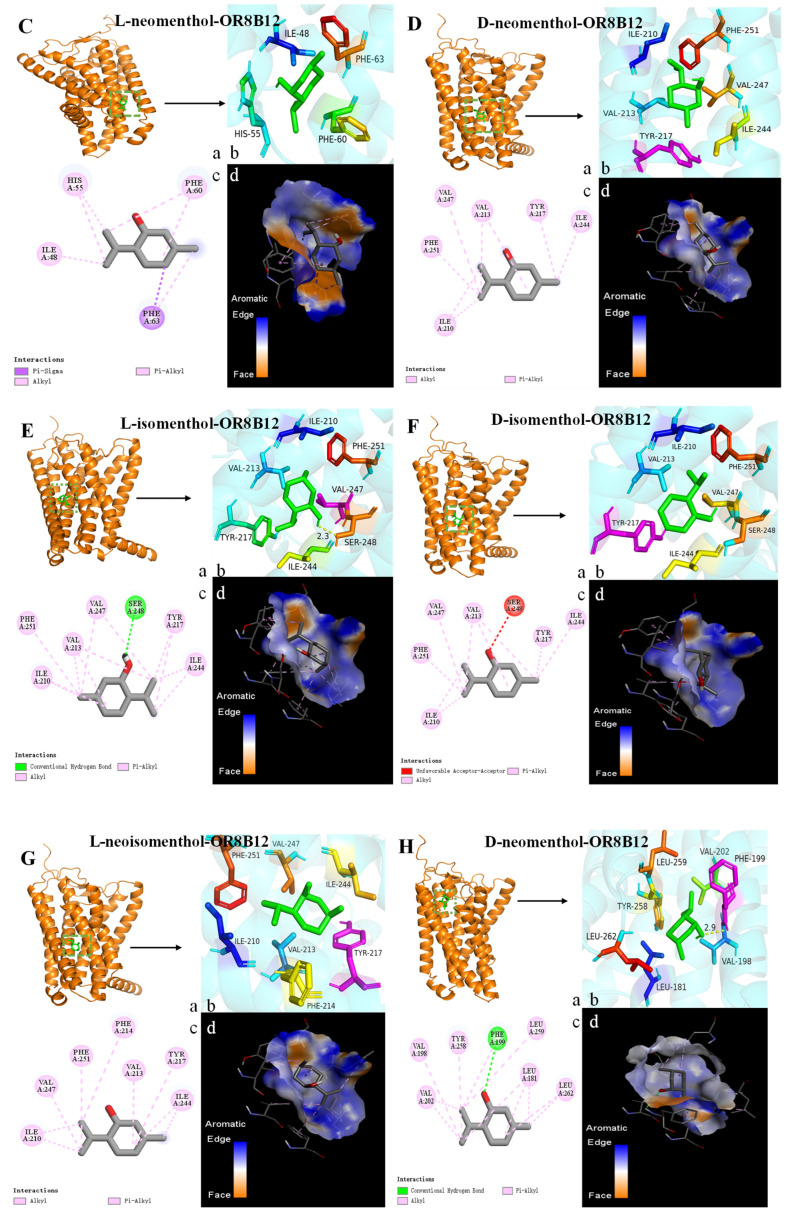
Molecular docking results of the olfactory receptor OR8B12 with eight menthol isomers. (**A**): L-menthol; (**B**): D-menthol; (**C**): L-neomenthol; (**D**): D-neomenthol; (**E**): L-isomenthol; (**F**): D-isomenthol; (**G**): L-neoisomenthol; (**H**): D-neoisomenthol. a–c: The global, local enlarged, and 2D plots of the docking. d: Binding pocket.

**Figure 6:**
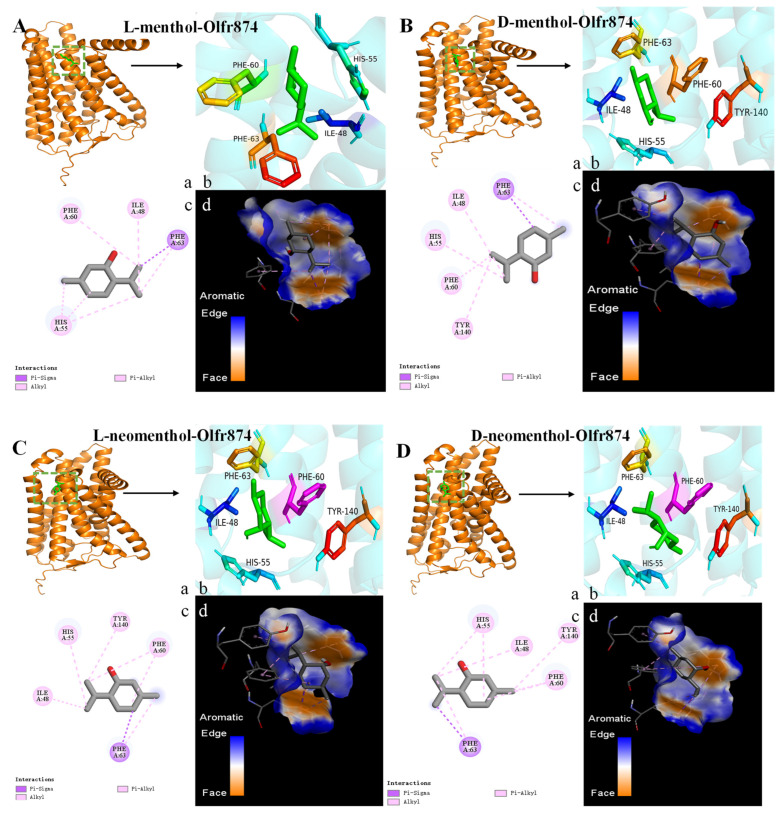

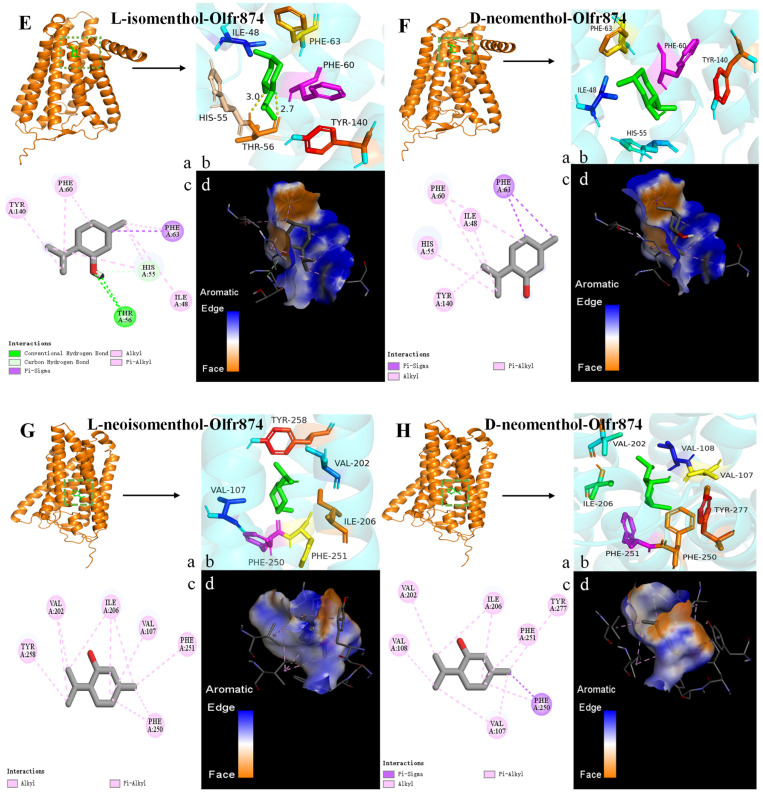
Molecular docking results of the olfactory receptor Olfr874 with eight menthol isomers. (**A**): L-menthol; (**B**): D-menthol; (**C**): L-neomenthol; (**D**): D-neomenthol; (**E**); L-isomenthol; (**F**): D-isomenthol; (**G**): L-neoisomenthol; (**H**): D-neoisomenthol. a–c: The global, local enlarged, and 2D plots of the docking. d: Binding pocket.

The diversity of amino acid sequences forms the structural basis for their binding to various odorants [48]. As shown in Figure 4A–H, Figure 5A–H and Figure 6A–H, key residues involved in menthol isomer binding to olfactory receptors were identified, such as Thr-57 (OR8B8), Leu-55 (OR8B8), Tyr-94 (OR8B8), Phe-199 (OR8B12), Ser-248 (OR8B12), His-55 (Olfr874), and Thr-56 (Olfr874). Some of the key amino acid residue positions matched the information reported in the literature: that is, regions TM3-TM7 are the binding sites for aroma compounds [49]. Interestingly, in addition to the conventional binding regions reported in the literature, some amino acid residues in the extracellular loop region were also involved in the interaction of menthol isomers with olfactory receptors in this study. In other words, a part of the amino acid residues in the extracellular loop region (His-55, His-56, Thr-56, Phe-168, Leu-181, etc.) was found to play an equally important role in the formation of hydrogen bonding and hydrophobic interactions in the present study.

#### 3.3.2. Comparison of Binding Energy Between Different ORs and Menthol Isomers

A binding energy of less than 0 is an important indicator of the spontaneous occurrence of molecular interactions [7]. In biology, such spontaneous interactions often involve molecular recognition and binding processes, such as protein–ligand interactions [50]. In the present work, there are significant differences in binding energy between the three olfactory receptors and menthol isomers, with binding energy ranging from −7.3 (OR8B8_D-menthol) to −5.1 (Olfr874_L-menthol) kcal/mol (Table 1).

Specifically, the binding energies of D-menthol, L-neoisomenthol, and D-neoisomenthol with human receptor OR8B8 are −7.3, −6.7, and −7.1 kcal/mol, respectively; with human receptor OR8B12, they are −5.4, −5.6, and −5.6 kcal/mol, respectively (Table 1). This indicates that the binding energies of the same compounds differ when binding to different receptors. It has been reported that Olfr874 is the specific receptor for L-menthol. By docking with eight isomers of menthol, OR8B8 has a higher affinity for menthol isomers than OR8B12 (the average binding energies are −6.1 and −5.45 kcal/mol, respectively). OR8B8 has a stronger affinity than OR8B12, and OR8B8 may be the human olfactory receptor with the higher homology to Olfr874.

However, as shown in Figure 2 and Table 1, binding energy is not an absolute factor in determining threshold levels. For example, D-menthol has the strongest affinity for the olfactory receptor OR8B8 (−7.3 kcal/mol), and its detection threshold is also the lowest among the eight isomers (4.734 mg/L). However, the binding energy between D-neoisomenthol and OR8B8 is lower than that between L-menthol and OR8B8. Nevertheless, the detection threshold of D-neoisomenthol (8.972 mg/L) is higher than that of L-menthol (5.166 mg/L), indicating that D-neoisomenthol binds more tightly to the OR8B8 receptor at the molecular level than L-menthol. Nevertheless, the activation of olfactory receptors depends not only on the binding of ligands to receptors but also on the complex post-receptor signaling processes. The binding affinity of D-neoisomenthol to the OR8B8 receptor is lower than that of L-menthol. Based solely on binding affinity, the detection threshold for D-neoisomenthol should be lower than that for L-menthol. However, the activation of olfactory receptors by odorants is only part of odor perception. There may be situations where certain olfactory receptors are selectively activated or downstream olfactory transduction signals are blocked. This could also result in a higher detection threshold for D-neoisomenthol compared to L-menthol [51]. Additionally, while L-menthol has relatively weak binding affinity with Olfr874 (−5.1 kcal/mol), it has the lowest detection threshold (5.166 mg/L) compared to other isomers. This may be because, although L-menthol exhibits weaker binding energy after binding to olfactory receptors, it may form a highly complementary conformation with the active site of Olfr874, inducing specific conformational changes in the receptor (such as the activated state of G-protein-coupled receptors). Other isomers, due to spatial steric hindrance or differences in polarity distribution, cannot effectively trigger such conformational changes. Additionally, odorants must possess certain molecular characteristics to provide more distinct and accurate sensory properties [52].

Additionally, as shown in Figure 2 and Table 1, the binding energy of D-menthol with OR8B8 (−7.3 kcal/mol) is significantly lower than that of L-menthol (−5.7 kcal/mol). Binding energy typically reflects the strength of the ligand–receptor interaction; a lower binding energy indicates more stable binding between the ligand and receptor. However, sensory evaluation results show that the detection thresholds of the two are similar (4.734 vs. 5.166 mg/L). We speculate that this may be related to receptor expression levels. Even though D-menthol has a lower binding energy with OR8B8, if the expression level of the OR8B8 receptor in olfactory receptor cells is low, the actual number of receptors available to bind with D-menthol may be insufficient to significantly lower its detection threshold. In contrast, although L-menthol has a higher binding affinity, if its corresponding receptor expression levels are higher or other compensatory mechanisms exist, its detection threshold may also be similar to that of D-menthol [53].

Comprehensive analyses showed that different olfactory receptors have different binding affinities for the same odorant. The average binding energy values of L-menthol, D-menthol, L-neomenthol, D-neomenthol, L-isomenthol, D-isomenthol, L-neoisomenthol, and D-neoisomenthol with the receptors (Olfr874, OR8B8, and OR8B12) were −5.43, −6.07, −5.37, −5.23, −5.7, −5.5, −6.2, and −6.37 kcal/mol, respectively. L-neomenthol and D-neomenthol exhibit lower affinity for the three olfactory receptors, with binding energies ranging from −5.2 to −5.6 kcal/mol. L-isomenthone and D-isomenthone also bind to Olfr874 (−5.7, −5.6 kcal/mol), OR8B8 (−5.8, −5.4 kcal/mol), and OR8B12 (−5.6, −5.5 kcal/mol), but with lower affinity. The docking results show that all odor molecules interact with surrounding amino acid residues via hydrophobic interactions, as shown in Figure 4, Figure 5 and Figure 6 (A–H), with detailed data reported in Table 2.

The eight menthol isomers exhibit different binding energies when bound to the same olfactory receptors. For example, the binding energies of OR8B8 with L-menthol, D-menthol, L-neomenthol, D-neomenthol, L-isomenthol, D-isomenthol, L-neoisomenthol, and D-neoisomenthol were −5.7, −7.3, −5.6, −5.2, −5.8, −5.4, −6.7, and −7.1 kcal/mol, respectively. The average binding energy between L-menthol and the three olfactory receptors is −5.43 kcal/mol. The average binding energy between D-menthol and the three olfactory receptors is lower than that of L-menthol, at −6.07 kcal/mol. Surprisingly, the average binding energies of L-neoisomenthol and D-neoisomenthol with the three olfactory receptors are relatively low among the eight isomers, yet their aroma intensities are not the strongest. This may be related to odor antagonism, thereby affecting the activity of olfactory neurons and altering human perception of odors [54]. The different isomers vary in spatial structure; thus, the binding sites and forces with the receptor proteins are different, and these differences further affect the combination of the odorant and the receptor.

In this study, although L-menthol and D-menthol do not have the strongest affinity for olfactory receptors, they have relatively low detection thresholds among the eight isomers. Therefore, we speculate that these two isomers may have higher receptor activation efficiency, thereby enhancing olfactory signal transmission and causing stronger olfactory perception. It has also been shown that each olfactory receptor binds aroma compounds with different affinities and produces different neuronal activities [55].

Furthermore, the binding energy between L-neomenthol and OR8B12 is −5.6 kcal/mol, which is higher than the binding energy between this compound and the other two receptors (OR8B8 and Olfr874), and it is also higher than the binding energy between most olfactory receptors and menthol isomers. This indicates that L-neomenthol has a weaker affinity for OR8B12. As shown in Table 2, L-menthol can only form hydrogen bonds with OR8B8, with a binding energy of −5.7 kcal/mol.

#### 3.3.3. Analyzing the Binding Forces and Sites Between ORs and Menthol Isomers

Binding energies and binding sites vary between different menthol isomers and olfactory receptors. L-menthol, L-neomenthol, L-isomenthol, and D-isomenthol all stably bind to the olfactory receptor OR8B8 through specific hydrogen bonding interactions, as well as hydrophobic interactions. Specifically, the four menthol isomers form hydrogen bonds with amino acid residues Thr-57, Tyr-94, Leu-55, and Thr-57 in OR8B8, respectively (Figure 4A,C,E,F and Table 2). For OR8B8, L-isomenthol facilitates the further enhancement of its binding stability by forming hydrogen bonds with Ser-248, D-neoisomenthol, and Phe-199 (Figure 5E,H and Table 2). Similarly, L-isomenthol enhanced its affinity by forming hydrogen bonds with His-55 and Thr-56 in Olfr874 (Figure 6E and Table 2). However, the spatial conflict between the Ser-248 residue in OR8B12 and the boundaries of the binding pockets of L-menthol and D-isomenthol is not favorable for the binding of these two menthol isomers, which may be responsible for the reduced binding energies (Figure 5A,F). From a molecular structural point of view, each isomer of menthol has a cyclic structure. This structure allows them to undergo π–π stacking with amino acid residues. In addition, the difference in the spatial positions of the isomers can also weaken the interaction between them due to the occurrence of spatial site resistance. Notably, researchers have also recently found that π–π stacking is an important driver of receptor interactions with aromatic compounds [56].

Table 2 reveals the key residues for the binding of eight menthol isomers to the olfactory receptor, such as His-55 (Olfr874_L-isomenthol), Thr-56 (Olfr874_L-isomenthol), Thr-57 (OR8B8_L-menthol) Tyr-94 (OR8B8_L-neomenthol), Leu-55 (OR8B8_L-isomenthol), Thr-57 (OR8B8_D-isomenthol), Ser-248 (OR8B12_L-isomenthol), and Phe-199 (OR8B12_D-Neoisomenthpl). In addition to hydrogen bonds, olfactory receptors rely primarily on hydrophobic interactions between the two to maintain the stability of the complex. Specifically, among all isomers, L/D-neoisomenthol showed the highest binding energy with the olfactory receptor; in addition to hydrogen bonding, it interacted with amino acid residues through hydrophobic forces, suggesting that hydrophobic forces play an important role in stabilizing the binding of the olfactory receptor to small molecules. It is worth noting that OR8B8 formed the most hydrogen bonds with eight isoforms, with an average binding energy of −6.1 kcal/mol, which is lower than that of OR8B12 (average binding energy of −5.45 kcal/mol) and Olfr874 (average binding energy of −5.65 kcal/mol).

Surprisingly, L-neoisomenthol was able to form hydrogen bonds with OR8B12 but not with OR8B8 and Olfr874. This finding reveals a subtle and complex dimension of the olfactory recognition mechanism: That is, the same aroma small molecule may bond differently with different olfactory receptors. This may be a result of differences in the structure of the olfactory receptors and the nature of amino acid residues. Although hydrogen bonding promotes binding and enhances affinity between olfactory receptors and aroma compounds, it cannot be simply concluded that the greater the number of hydrogen bonds, the greater the binding energy. This finding suggests that the interactions between olfactory receptors and aroma compounds are far more complex than a single hydrogen bonding interaction. In addition to hydrogen bonding, there are multiple driving forces, such as hydrophobic interactions and π–π stacking between olfactory receptors and aroma compounds.

As shown in Table 2, this study reveals complex interactions between menthol isomers and olfactory receptors Olfr874, OR8B8, and OR8B12. Specifically, all six menthol isomers (L/D-menthol, L/D-neomenthol, and L/D-isomenthol) were able to form hydrophobic interactions with Ile-48, His-55, Phe-60, and Ph-63 in Olfr874. Surprisingly, L-menthol did not form a hydrophobic interaction with Tyr-140. The binding regions of L-neoisomenthol and D-neoisomenthol to olfactory receptors differ from those of the other menthol isomers. Their binding sites to the three olfactory receptors are mainly concentrated in the TM5-TM6 region. These interactions are critical for understanding how menthol is recognized by the olfactory system. For example, D-menthol interacts with nine amino acid residues in OR8B8 (Leu-105, Val-108, Val-109, Val-203, Ile-207, Phe-251, Phe-252, Tyr-259, and Tyr-278), and L-neoisomenthol interacts with nine hydrophobic amino acid residues in OR8B8 (Leu -105, Val-108, Val-109, His-159, Val-203, Ile-207, Phe-251, Phe-252, and Tyr-259) (Table 2). Although both have the same number of amino acid residues interacting with the same olfactory receptor, the binding energies produced differ due to the different amino acid species. This suggests that the amino acid residues and interactions produced by different menthol isomers when bound to the same olfactory receptor are also different. The docking results show that the amino acid residues that produce hydrogen bonding or hydrophobic interactions between menthol isomers and olfactory receptors are closely positioned together, forming a relatively intact hydrophobic region, which provides an ideal environment for their binding [57,58]. This binding is not only stable but it is also essential for the olfactory recognition process of menthol, and through these interactions, menthol isomers are able to efficiently activate the olfactory receptor, which triggers our perception of a minty aroma.

It has been shown that amino acid residues with a benzene ring structure are more likely to form hydrophobic interactions with aromatic compounds, which enhances the binding capacity of olfactory receptors to ethyl vanillin [46]. In the present study, amino acid residues containing a benzene ring structure (e.g., Phe-250 and Tyr-258 in Olfr874, Phe252 and Tyr-278 in OR8B8, and Phe-251 and Tyr-217 in OR8B12) tended to form hydrophobic interactions with menthol isomers. In addition to hydrophobic interactions, the amino acid residues of the benzene ring structure also participate in π–π stacking interactions, which further enhance the affinity with aroma compounds. In addition, the recurrence of certain amino acid residues reveals their importance in the binding of menthol isomers, which are closely related to the production of minty aroma; for example, residues Ile-48, His-55, Phe-60, Phe-63, and Tyr-140 in the Olfr874 receptor; His-56, Phe-61, and Tyr-64 in the OR8B8 receptor; and Ile-210, Val-213, Tyr-217, Ile-244, Val-247, and Phe-251 in the OR8B12 receptor. Based on these key residues, virtual screening techniques can be applied to identify aroma compounds with specific binding properties and thus build a comprehensive database of minty aroma-related compounds.

## 4. Conclusions

In the present study, the odor characteristics and detection thresholds of eight menthol isomers were examined, along with the mechanisms of their perception. The results show that the odor intensity and recognition of the menthol isomers increase with increasing concentration, but the different isomers significantly differ in their odor characteristics, which might be related to their spatial structures and intermolecular interactions. Calculating the docking energies and analysis of the binding sites reveals that the menthol isomers varied in their affinity with olfactory receptors. These findings provide molecular-level insights into the odor perception mechanism of menthol isomers and provide a scientific basis for the interaction of menthol isomers with odor receptors. In reality, olfactory perception involves a series of complex physiological processes. Future studies are necessary to focus on the conformational relationships of menthol isomers with other terpenoids and to reveal the potential effects of key residue changes by combining mutant docking simulations and cellular validation approaches.

## Figures and Tables

**Table 1:** Summary of the binding energy between olfactory receptors and ligands.

Ligands	Binding Energy (kcal/mol)		
	Olfr874	OR8B8	OR8B12
L-menthol	−5.1	−5.7	−5.5
D-menthol	−5.5	−7.3	−5.4
L-Neomenthol	−5.3	−5.6	−5.2
D-Neomenthol	−5.3	−5.2	−5.2
L-Isomenthol	−5.7	−5.8	−5.6
D-Isomenthol	−5.6	−5.4	−5.5
L-Neoisomenthol	−6.3	−6.7	−5.6
D-Neoisomenthol	−6.4	−7.1	−5.6

**Table 2:** Summary of the binding forces and sites between olfactory receptors and ligands.

Receptor	Ligand	Hydrogen Bonding Amino Acid Residues	Hydrophobic Amino Acid Residues
Olfr874	L-Menthol	-	Ile-48, His-55, Phe-60, Phe-63
	D-Menthol	-	Ile-48, His-55, Phe-60, Phe-63, Tyr-140
	L-Neomenthol	-	Ile-48, His-55, Phe-60, Phe-63, Tyr-140
	D-Neomenthol	-	Ile-48, His-55, Phe-60, Phe-63, Tyr-140
	L-Isomenthol	His-55 Thr-56	Ile-48, His-55, Phe-60, Phe-63, Tyr-140
	D-Isomenthol	-	Ile-48, His-55, Phe-60, Phe-63, Tyr-140
	L-Neoisomenthol	-	Val-107, Val-202, Ile-206, Phe-250, Phe-251, Tyr-258
	D-Neoisomenthol	-	Val-107, Val-108, Val-202, Ile-206, Phe-250, Phe-251, Tyr-277
OR8B8	L-Menthol	Thr-57	His-56, Phe-61, Tyr-64
	D-Menthol	-	Leu-105, Val-108, Val-109, Val-203, Ile-207, Phe-251, Phe-252, Tyr-259, Tyr-278
	L-Neomenthol	Tyr-94	Phe-8, Val-9, Phe-168
	D-Neomenthol	-	Phe-8, Val-9, Tyr-94, Phe-168
	L-Isomenthol	Leu-55	Ile-49, Arg-50, His-56, Phe-61, Tyr-64
	D-Isomenthol	Thr-57	Ile-49, His-56, Phe-61, Tyr-64
	L-Neoisomenthol	-	Leu-105, Val-108, Val-109, His-159, Val-203, Ile-207, Phe-251, Phe-252, Tyr-259
	D-Neoisomenthol	-	Ile-49, Arg-50, His-56, Phe-61, Tyr-64
OR8B12	L-Menthol	-	Ile-210, Val-213, Tyr-217, Ile-244, Val-247, Ser-248, Phe-251
	D-Menthol	-	Ile-210, Val-213, Tyr-217, Ile-244, Val-247, Phe-251
	L-Neomenthol	-	Ile-48, His-55, Phe-60, Phe-63
	D-Neomenthol	-	Ile-210, Val-213, Tyr-217, Ile-244, Val-247, Phe-251
	L-Isomenthol	Ser-248	Ile-210, Val-213, Tyr-217, Ile-244, Val-247, Phe-251
	D-Isomenthol	-	Ile-210, Val-213, Tyr-217, Ile-244, Val-247, Ser-248, Phe-251
	L-Neoisomenthol	-	Ile-210, Val-213, Phe-214, Tyr-217, Ile-244, Val-247, Phe-251
	D-Neoisomenthol	Phe-199	Leu-181, Val-198, Val-202, Tyr-258, Leu-259, Leu-262

## Data Availability

The original contributions presented in this study are included in the article. Further inquiries can be directed to the corresponding authors.
